# Localization to delocalization probed by magnetotransport of hBN/graphene/hBN stacks in the ultra-clean regime

**DOI:** 10.1038/s41598-021-98266-4

**Published:** 2021-09-22

**Authors:** Takuya Iwasaki, Satoshi Moriyama, Nurul Fariha Ahmad, Katsuyoshi Komatsu, Kenji Watanabe, Takashi Taniguchi, Yutaka Wakayama, Abdul Manaf Hashim, Yoshifumi Morita, Shu Nakaharai

**Affiliations:** 1 International Center for Young Scientists, National Institute for Materials Science (NIMS), Tsukuba, Ibaraki 305-0044 Japan; 2 International Center for Materials Nanoarchitectonics, NIMS, Tsukuba, Ibaraki 305-0044 Japan; 3 Malaysia-Japan International Institute of Technology, Universiti Teknologi Malaysia, Jalan Sultan Yahya Petra, 54100 Kuala Lumpur, Malaysia; 4 Research Center for Functional Materials, NIMS, Tsukuba, Ibaraki 305-0044 Japan; 5 Faculty of Engineering, Gunma University, Kiryu, Gunma 376-8515 Japan; 6 Present Address: Department of Electrical and Electronic Engineering, Tokyo Denki University, 5 Senju-Asahi-cho, Adachi-ku, Tokyo, 120-8551 Japan

**Keywords:** Electronic and spintronic devices, Electronic properties and materials

## Abstract

We report on magnetotransport in a high-quality graphene device, which is based on monolayer graphene (Gr) encapsulated by hexagonal boron nitride (hBN) layers, i.e., hBN/Gr/hBN stacks. In the vicinity of the Dirac point, a negative magnetoconductance is observed for high temperatures >  ~ 40 K, whereas it becomes positive for low temperatures ≤  ~ 40 K, which implies an interplay of quantum interferences in Dirac materials. The elastic scattering mechanism in hBN/Gr/hBN stacks contrasts with that of conventional graphene on SiO_2_, and our ultra-clean graphene device shows nonzero magnetoconductance for high temperatures of up to 300 K.

## Introduction

Magnetotransport is a sensitive probe for quantum interference of electron wavefunctions. As a result of scattering with impurities, electrons could return to the original place and form a closed trajectory. The probability of this event can be enhanced by quantum interference within a closed trajectory due to time-reversal symmetry. This phenomenon is known as Anderson localization (AL) and its precursor, weak localization (WL) has also been studied for a long time^1–4^. The time-reversal symmetry breaking by, for example, applying a magnetic field causes destructive interference, which is observed as negative magnetoresistance^5,6^.

AL in Dirac materials has long been studied (e.g., see Refs.^7,8^); this interest was sparked by the discovery of graphene (a textbook example of a Dirac material) on SiO_2_^9^, and these studies on the early stage were summarized in a review^10^. In particular, the WL in graphene has attracted attention due to its distinctive features. In graphene, the backscattering of electrons is suppressed due to the Berry phase of π^11^. In other words, the “chirality” and conservation of associated pseudospin due to the equivalence of two sublattices inhibits backscattering. Because of the suppression of backscattering probability, the interference of charge carriers in graphene leads to “delocalization”^12^. This effect is assigned to weak antilocalization (WAL), and experimentally observed as positive/negative magnetoresistance/conductance, respectively. However, the interference effects in graphene depend on not only phase-breaking inelastic scattering, but also several types of elastic scattering. One is intravalley scattering, which leads to the cancellation of WAL. Another is intervalley scattering, which mixes the states of two valleys, K and K’. The mixing of opposite valley states negates the intravalley effects, resulting in the occurrence of WL in graphene^12^.

Magnetotransport and localization effects have been investigated experimentally in ultrathin graphite grown on silicon carbide^13^ or graphene flakes on SiO_2_^14–19^. In particular, Tikhonenko et al. reported that the magnetoconductance (MC) in graphene shows positive values for low temperatures, i.e., WL, while the MC is negative for high temperatures, i.e., WAL^18^. The crossover between WL and WAL is fixed by the competition between inelastic phase coherence time ($\tau_{\phi }$) and two elastic scattering times, intravalley ($\tau_{*}$) and intervalley ($\tau_{{\text{i}}}$). When $\tau_{\phi } < \tau_{{\text{i}}} ,\tau_{*}$ is on the higher temperature side, the quantum interference tends to WAL, while the opposite condition leads to WL; the former was observed up to a temperature of 204 K^18^, which contradicts conventional wisdom. Such phase coherence at a high temperature was attributed to sufficiently weak electron–phonon coupling, while electron–electron interaction was pointed out as a main factor of dephasing in a high temperature regime^18^.

With the advent of hexagonal boron nitride (hBN) and the development of fabrication technology, the quality of graphene devices, e.g., hBN/graphene(Gr)/hBN stacks and superlattices, has been dramatically improved^20–28^. Here, it is noted that phase coherent transport phenomena have been observed up to high temperatures^23,24^. Even though ultra-clean hBN/Gr/hBN devices have so far been studied, systematic MC remains unexplored.

In this study, we investigate the MC and quantum interference-induced localization effect in ultra-clean hBN/Gr/hBN stacks for a wide temperature range of between 6 and 300 K. A high-quality device is fabricated based on a monolayer graphene encapsulated with hBN layers by an all-dry transfer technique^23,24^.

**Figure 1:**
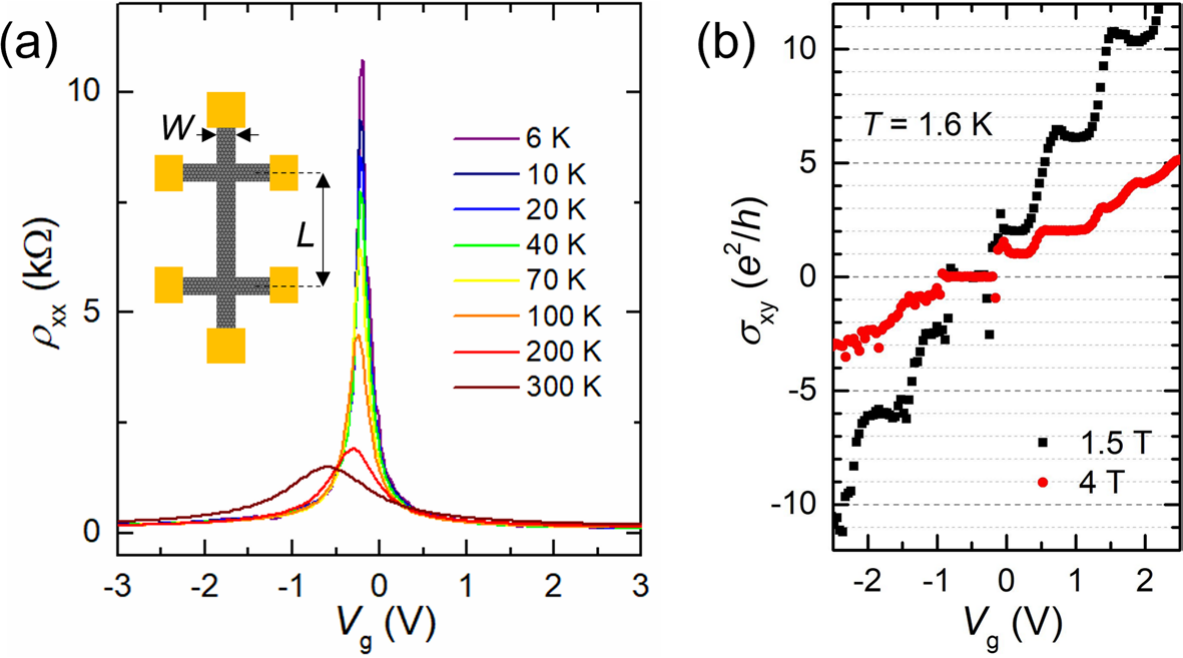
(**a**) Longitudinal resistivity of the device as a function of back-gate voltage for various temperatures. Inset: a schematic illustration of the Hall-bar channel geometry. The symbols *L* and *W* indicate the channel length and width, respectively. (**b**) Hall conductivity of the device as a function of back-gate voltage at *T* = 1.6 K. The black square corresponds to the data at *B* = 1.5 T, and the red circle at *B* = 4 T.

Firstly, we characterize the quality of our device. Figure 1a shows the longitudinal resistivity (*ρ*_xx_) as a function of the back-gate voltage (*V*_g_) for various temperature (*T*). Except at *T* = 300 K, the Dirac point (DP) at *V*_g_ = −0.2 V indicates that the influence from charged impurities is extremely small. At *T* = 300 K, the DP slightly shifts to a *V*_g_ ~ −0.6 V, which can be ascribed to the influence of charged impurities in the substrate. By following the method described in Ref.^29^, the residual carrier density at *T* = 6 K is estimated to be *n*_res_ ~ 1.6 × 10^10^ cm^−2^, which is smaller than the typical carrier density of ~ 10^11^ cm^−2^ in graphene on SiO_2_^30^ (further analysis is shown in Supplementary Information S3, Fig. S5). Moreover, the mobility at *T* = 6 K is *μ*_h_ ~ 200,000 cm^2^V^−1^ s^−1^ for holes and *μ*_e_ ~ 130,000 cm^2^V^−1^ s^−1^ for electrons, which is comparable to recent high-quality graphene devices^22^. These results indicate the ultra-clean condition in our device. Note that for the localization studies, the highest mobility was ~ 12,000 cm^2^V^−1^ s^−1^ in the graphene/SiO_2_ devices^18^, and ~ 30,000–120,000 cm^2^V^−1^ s^−1^ in the graphene/hBN devices^31^.

Furthermore, the quality of our device can be confirmed from the quantum Hall effect under a perpendicular magnetic field (*B*) shown in Fig. 1b. At *T* = 1.6 K, the Hall conductivity (*σ*_xy_) shows well-resolved plateaus at *B* = 1.5 T. These plateaus are located at *σ*_xy_ = *νe*^2^/*h*, (where *ν* =  ± *g*_s_*g*_v_(|*N*_LL_|+ 1/2) is the filling factor, *g*_s_, *g*_v_ = 2 are spin and valley degeneracy, respectively, *N*_LL_ is the Landau level index, *e* the elementary charge, and *h* Planck’s constant); proving that the device consists of a monolayer graphene^32^. Moreover, the additional plateaus appear for *ν* =  ± 1, + 3, and + 4 at *B* = 4 T, indicating spin/valley degenerated Landau level splitting. Such a broken symmetry property requires an extremely high magnetic field for graphene on SiO_2_^33^.

**Figure 2:**
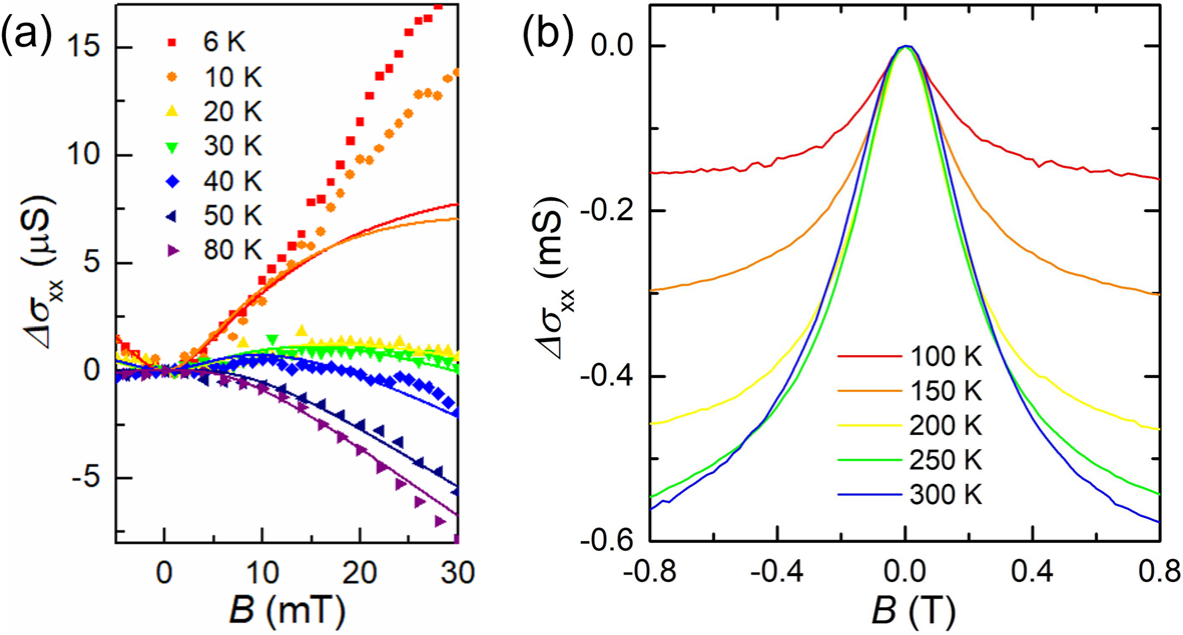
(**a**) Magnetoconductance of the device at the DP for the low temperature magnetic field regime. The symbols show the experimental data, while the solid lines represent the fitting results using Eq. (1). (**b**) Magnetoconductance for the relatively high temperature, magnetic field regime. The lines correspond to the experimental data.

Next, we discuss the MC of our device. Here we define MC as *Δσ*_xx_(*B*) = *σ*_xx_(*B*) − *σ*_xx_(0), and the gate voltage is fixed at the DP. To analyze the localization effects due to quantum interference, the quantum correction theory is applied to the MC as shown in the following formula^12^:

1 $$\begin{aligned} \Delta \sigma_{{{\text{xx}}}} \left( B \right) & = \frac{{e^{2} }}{\pi h}\left[ {F\left( {\frac{{\tau_{B}^{ - 1} }}{{\tau_{\phi }^{ - 1} }}} \right) - F\left( {\frac{{\tau_{B}^{ - 1} }}{{\tau_{\phi }^{ - 1} + 2\tau_{{\text{i}}}^{ - 1} }}} \right) - 2F\left( {\frac{{\tau_{B}^{ - 1} }}{{\tau_{\phi }^{ - 1} + \tau_{{\text{i}}}^{ - 1} + \tau_{*}^{ - 1} }}} \right)} \right], \\ F\left( z \right) & = \ln \left( z \right) + \psi \left( {0.5 + z^{ - 1} } \right) \\ \end{aligned}$$

where, $\tau_{B}^{ - 1} = 4eDB/\hbar$, *D* = *v*_F_*l*_mfp_/2 is a diffusion coefficient (*v*_F_ = 10^6^ m/s is the Fermi velocity of graphene, *l*_mfp_ = *h*/(2*e*^2^*ρ*_xx_ $\sqrt {\pi n}$) is the mean free path), *ℏ* = *h*/(2π), and $\psi$ is the digamma function. The first term of Eq. (1) corresponds to the effect of WL, i.e., positive MC, which becomes dominant when *τ*_i_^–1^ and τ_*_^–1^ are large. On the other hand, the second and third terms reflect the effect of WAL, which is dominant when *τ*_i_^–1^ and τ_*_^–1^ are small. The intervalley scattering characterized with *τ*_i_^–1^ is caused by atomically sharp defects, such as graphene edges. τ_*_^–1^ is a parameter that characterizes the combined effect including intravalley scattering, Fermi surface anisotropy (trigonal warping), and an effective random magnetic field due to defects with a lattice spacing size, dislocation, and ripple. In the following, we set *n* = *n*_res_ and estimated the “mean free path at the DP”, which characterizes the length scale in the vicinity of the DP. Figure 2a shows the MC for *T* ≤ 80 K and *B* ≤ 30 mT. We should focus on the regime where the magnetic length *l*_B_ = $\sqrt{\hbar/({eB})}$ is longer than the mean free path (*l*_mfp_ $\ll$ *l*_B_). The mean free path of our device is *l*_mfp_ ~ 50–160 nm in the vicinity of DP for the all-*T* range. Therefore, we analyzed the MC fitting in the range of *B* ≤ 30 mT (*l*_B_ ≥ 150 nm). In this low *B* region, the positive MC for *T* ≤ 30 K suggests that WL is dominant in the interference. The MC at *T* = 40 K is positive in the low *B* region, and the sign of the curve coefficient is inverted to a negative value in the relatively large *B* region. The magnetic field where the sign is inverted corresponds to a value of the intervalley scattering rate in a magnetic field scale *B*_i_ = *τ*_i_^–1^*ℏ*/(4*De*). When the intravalley scattering effect is negligible (τ_*_^–1^ ~ *τ*_i_^–1^), the sign is fully inverted in the large *B* region. On the other hand, when the intravalley scattering effect is strong (τ_*_^–1^ $\gg$ *τ*_i_^–1^), the sign is not inverted even in the large *B* region. This corresponds to the fact that the intravalley scattering destroys the interference within one valley, and the intervalley scattering mixes the state within two valleys and causes the interference. We confirmed that for *T* < 6 K the MC fitting deviation is pronounced and the resistivity at the DP becomes saturated (not shown). This is attributed to the influence of electron/hole puddles when their energy scale exceeds the thermal energy at *T* ~ 6 K, which almost agrees with the Gr/hBN devices in the literature^20^. On the other hand, the MC is negative for *T* ≥  ~ 40 K. This indicates that the phase coherence time is suppressed, leading to WAL. The crossover between WL and WAL occurs at *T* ~ 40 K.

Figure 2b exhibits the MC for *T* ≥ 100 K and *B* ≤ 0.8 T. In this region, the MC has nonzero values even at *T* = 300 K, in contrast to the results of previous graphene/SiO_2_ devices under a different condition; for example, the MC almost disappears at *T* = 204 K^18^.

Here we comment on the fact that the electron and hole puddles can co-exist at the DP, where the magnetotransport might be described by the two-carrier model^14,34^. This classical model implies that the magnetoresistance behaves as *B*^2^^14^, which is also compatible with our devices in the high-temperature regime (detailed in Supplementary Information S1). Although our scenario gives a consistent picture from low- to high-temperature regime on an equal footing, it is left as a future work to fix the unique picture among several scenarios (e.g., two-fluid models, hydrodynamic picture) especially in the high-temperature regime.

**Figure 3:**
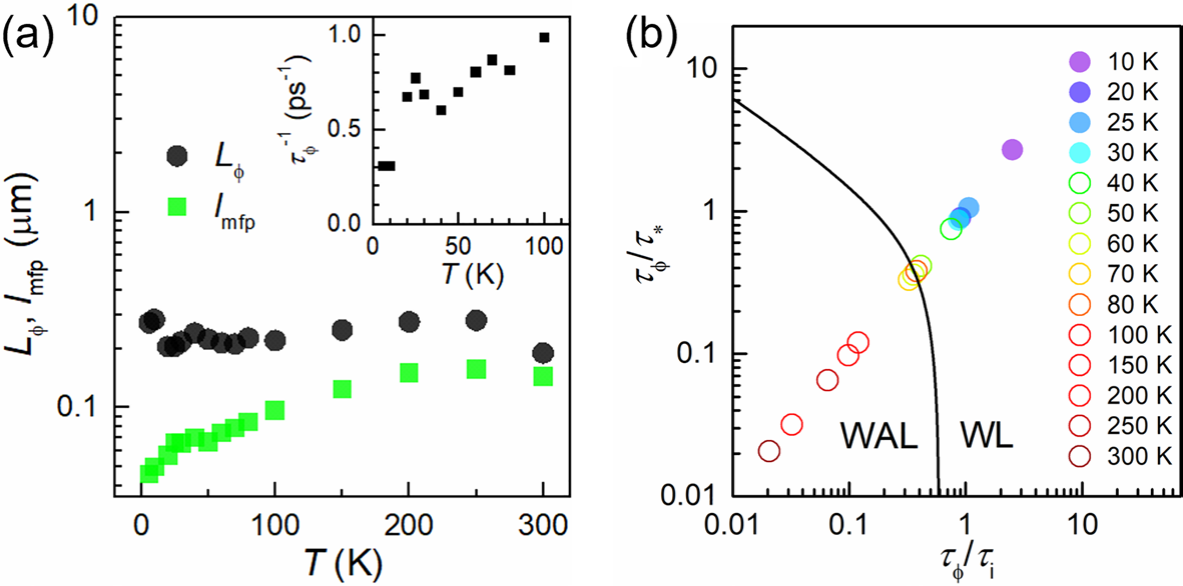
(**a**) Temperature dependence of characteristic lengths. The green squares represent the mean free path. The black circles present the phase coherence length estimated by fitting with Eq. (1). Inset: the temperature dependence of the phase-breaking rates estimated by the fitting with Eq. (1). (**b**) Phase diagram of quantum interference in graphene. The solid line is drawn along Eq. (2) with *Δσ*_xx_ = 0. If the sum of MC in the range of *B* < 30 mT is positive, the data are shown as full circles, otherwise the data are depicted as open circles.

Figure 3a shows the temperature dependence of the characteristic lengths responsible for the localization effect. The phase coherence length ($L_{\phi }$) is calculated from $L_{\phi } = \sqrt {D\tau_{\phi } }$, with $\tau_{\phi }$ obtained by the fitting of Eq. (1) to the MC. $\tau_{\phi }$ depends on temperature and is in the order of several picoseconds at the lowest temperature of our study. The mean free path in the vicinity of the DP is also shown, which is shorter than the phase coherence length, implying the diffusive picture in the vicinity of the DP. The inset of Fig. 3a depicts the temperature dependence of the phase-breaking scattering rate for *T* ≤ 100 K.

The crossover of WL/WAL can also be seen in the phase diagram in Fig. 3b that plots the ratio of phase coherence time to elastic scattering times. For low temperatures, two elastic scatterings predominate the quantum interference, leading to the WL effect (positive MC). On the other hand, it leads to WAL effect (negative MC) for high temperatures. The solid line is drawn along *Δσ*_xx_ = 0 in the following formula simplified from Eq. (1) when *z* $\ll$ 1 (at low *B*)^18^:

2 $$\Delta \sigma_{xx} = \frac{{e^{2} }}{24\pi h}\left( {\tau_{B}^{ - 1} \tau_{\phi } } \right)^{2} \left[ {1 - \frac{1}{{\left( {1 + 2\tau_{\phi } /\tau_{i} } \right)^{2} }} - \frac{2}{{\left( {1 + \tau_{\phi } /\tau_{i} + \tau_{\phi } /\tau_{*} } \right)^{2} }}} \right]$$

Although the results give qualitatively consistent picture in Fig. 3b, please note that the assumption *z* $\ll$ 1 does not always hold in the ‘phase boundary’ between WL and WAL regions and the boundary is not quantitative and rather just a guide for eyes.

Basically, there is no significant difference between intravalley and intervalley scattering rates in our study (see also Ref.^18^ for a comparison. Please note, however, that there is some ambiguity in the fitting procedure of intravalley and intervalley scattering rates). It is reasonable to think that key factors responsible for the scattering mechanism are severely limited in our high-quality device. For hBN/Gr/hBN stacks with a moderate mobility, intravalley scattering is a dominant factor for fixing the mobility, which can be due to random pseudomagnetic fields induced by strain fluctuations^35^. In our case, for hBN/Gr/hBN stacks with an ultra-high mobility, intravalley scattering is further reduced and $\tau_{*}$ becomes comparable with $\tau_{i}$, which implies the suppression of strain fluctuation due to the long-range nature of the corrugations in hBN/Gr/hBN stacks^36^. Note that we have also characterized magnetotransport at a different gate voltage near the DP, in which well-consistent results have been obtained (detailed in Supplementary Information S2, Figs. S3 and S4).

In conclusion, magnetotransport in ultra-clean hBN/Gr/hBN stacks has been investigated for a wide temperature range. Our result demonstrated an approach for weak localization effects in an ultra-clean setting. Meanwhile, strong localization effects are left to be studied as an alternative approach, for which a promising strategy is to introduce defects into graphene in a systematic way. For example, helium ion irradiation by an ion microscope with atomistic resolution is suited to this approach^37^, which was discussed in another paper^38^. Careful assignment of the scattering mechanism in normal (magneto) resistance of hBN/Gr/hBN moiré superlattices is a future problem^39,40^.

## Methods

The detailed device fabrication process is described in, for example, Ref.^23,24^. An hBN/Gr/hBN heterostructure on a heavily doped Si substrate with a 90-nm-thick SiO_2_ layer was firstly assembled by the all-dry transfer process^23,24,28^. The thicknesses of the top and bottom hBN layers are 25 nm and 34 nm, respectively. The stack was patterned into Hall bar geometry as schematically shown in the inset of Fig. 1a, using electron beam (EB) lithography and reactive ion etching. Then, one-dimensional edge contacts of Cr/Au were fabricated by EB lithography, EB deposition, and lift-off processes^21^. The channel length (*L*) and width (*W*) are 3.2 μm and 0.8 μm, respectively. The Si substrate was used as a back gate for tuning the Fermi energy. The four-terminal resistivity of the device was measured using AC lock-in techniques with an excitation current of 10 nA and a frequency of 17 Hz in a cryostat with a variable temperature insert, which controlled the temperature from 1.6 to 300 K. A magnetic field was applied perpendicularly to the substrate using a superconducting magnet.

## Supplementary Information

Supplementary Information.

## Data Availability

All data needed to evaluate the conclusions in the paper are present in the paper and/or the Supplementary Materials. Additional data related to this paper may be requested from the authors.
