# Supplementary material for: Localization to delocalization probed by magnetotransport of hBN/graphene/hBN stacks in the ultra-clean regime
Source: Sci Rep. 2021 Sep 22;11:18845. doi: 10.1038/s41598-021-98266-4 (PMC8458370; doi:10.1038/s41598-021-98266-4)
Supplement: Supplementary file 1 — Supplementary Information. [file 41598_2021_98266_MOESM1_ESM.pdf]

Supplementary Information:

"Localization to delocalization probed by magnetotransport of hBN/graphene/hBN stacks in the ultra-clean regime"

Takuya Iwasaki[1,2,*], Satoshi Moriyama[2,†,$], Nurul Fariha Ahmad[2,3], Katsuyoshi Komatsu[2], Kenji Watanabe[4], Takashi Taniguchi[2], Yutaka Wakayama[2], Abdul Manaf Hashim[3], Yoshifumi Morita[5,‡], and Shu Nakaharai[2,#]

[1]International Center for Young Scientists, National Institute for Materials Science (NIMS), Tsukuba, Ibaraki 305-0044, Japan.
[2]International Center for Materials Nanoarchitectonics, NIMS, Tsukuba, Ibaraki 305-0044, Japan.
[3]Malaysia-Japan International Institute of Technology, Universiti Teknologi Malaysia, Jalan Sultan Yahya Petra, 54100 Kuala Lumpur, Malaysia.
[4]Research Center for Functional Materials, NIMS, Tsukuba, Ibaraki 305-0044, Japan.
[5]Faculty of Engineering, Gunma University, Kiryu, Gunma 376-8515, Japan.

Corresponding authors.
*IWASAKI.Takuya@nims.go.jp, †moriyama.satoshi@mail.dendai.ac.jp,
‡morita@gunma-u.ac.jp, #NAKAHARAI.Shu@nims.go.jp

$Current affiliation: Department of Electrical and Electronic Engineering, Tokyo Denki University, 5 Senju-Asahi-cho, Adachi-ku, Tokyo 120-8551, Japan.

S1: More on the magnetotransport at the Dirac point
S2: Magnetotransport near the Dirac point: a comparative study
S3: Temperature dependence of the conductivity and resistivity

S1: More on the magnetotransport at the Dirac point

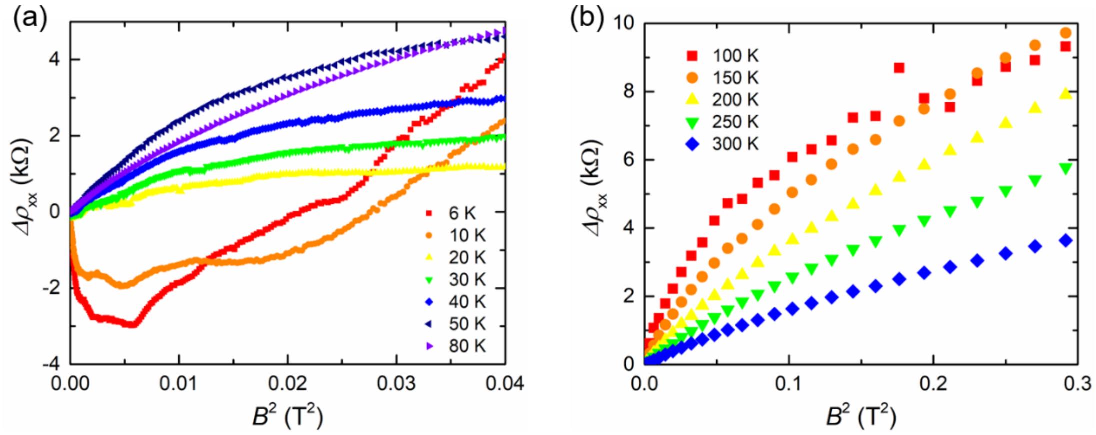

Figure S1. (a) Magnetoresistance at the Dirac point for the low temperature, low magnetic field regime. (b) Magnetoresistance for the relatively high temperature regime.

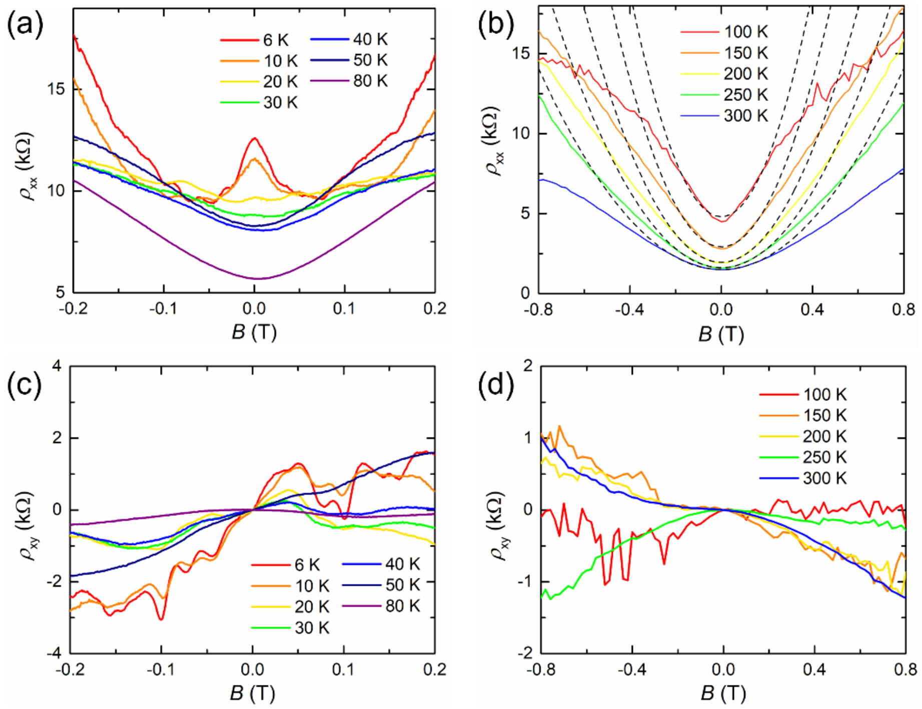

Figure S2. Hall measurement results of our device. (a) $\rho_{xx}(B)$ for $T \leq 80$ K. (b) $\rho_{xx}(B)$ for $T \geq 100$ K. The solid lines show the measurement results. The dashed lines represent the fitting results with the simple quadratic function. (c) $\rho_{xy}(B)$ for $T \leq 80$ K. (d) $\rho_{xy}(B)$ for $T \geq 100$ K.

In order to be complementary to the scenario in the main text, let us comment on the two-carrier model for the magnetoresistance (MR) at the Dirac point (DP) [1]. This model leads to MR, which behaves as $\Delta\rho_{xx} \propto B^2$ [1,2]. Figure S1 shows the $\Delta\rho_{xx}$ versus B^2 plot of our hexagonal boron nitride (hBN)/graphene (Gr)/hBN device at the DP for various temperatures. For the high temperature $T \geq 40$ K and low magnetic field regime $B^2 < 0.05$ T^2,

the MR becomes close to B^2 dependence. Moreover, we have analyzed the data in Figure S2 by the two-fluid model. Although the weak-(anti)localization (WAL) scenario describes our device from low- to high-temperature regime on an equal footing as discussed in the main text, we note that the two-carrier model behavior is also compatible with the above data in the high-temperature regime.

S2: Magnetotransport near the Dirac point: a comparative study

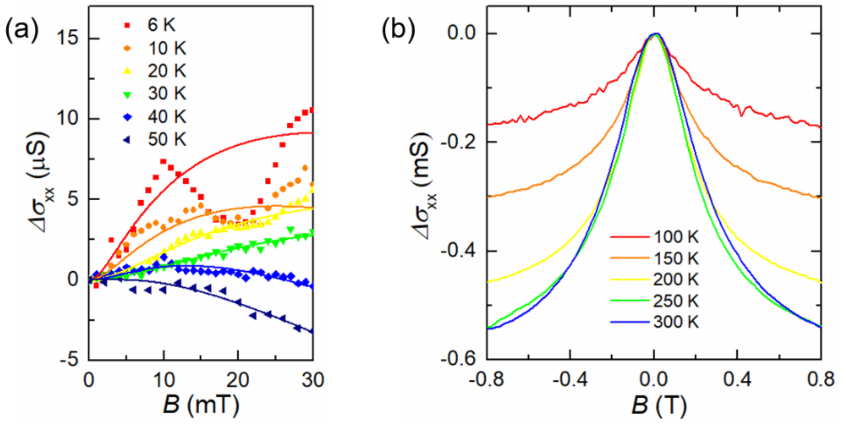

Figure S3. Magnetoconductance of the device at gate voltage V_g' = V_{DP} + 0.04 V. (a) For low temperature, magnetic field regime. The symbols represent the experimental data, while the solid lines represent the fitting results by Eq. (1) in the main text. (b) For the relatively high temperature magnetic field regime. The lines correspond to the experimental data.

For complementary analysis, we present the characterization of hBN/Gr/hBN device discussed in the main text at the different gate voltage V_g' = V_{DP} + 0.04 V, where the V_{DP} is the gate voltage at the DP. This corresponds to the electron density $n_e = 7.0 \times 10^9$ cm^{-2}, derived from $n_e = (C_g/e)|V_{DP} - V_g|$, where $C_g = \varepsilon_0\varepsilon/t$ is gate capacitance per unit area, ε_0 vacuum permittivity, ε = 3.9 the dielectric constant of SiO_2 and hBN, t = 124 nm the total thickness of the gate dielectric, and e the electron charge. From the Hall measurement results of the device at V_g' and T = 6 K, we estimate the electron mobility μ ~ 130,000 cm^{2}V^{-1}s^{-1}, mean free path l_{mfp} ~ 130 nm, and diffusion coefficient D ~ 630 cm^2/s (D ~ 230 cm^2/s at the DP).

Figure S3(a) shows the magnetoconductance (MC) for temperature $T \leq 50$ K and magnetic field $B \leq 30$ mT. Similar to the result at the DP in the main text, we observe positive MC for $T \leq 30$ K corresponding to the WL-dominant interference. At T = 6 K and 10 K, the well-fitted B range is limited due to quantum oscillation/fluctuation onset. On the other hand, there is the negative MC for $T \geq$ ~40 K, indicating WAL-dominant interference. The crossover between WL and WAL occurs at T ~ 40 K, which is consistent with the result in the

main text. Figure S3(b) exhibits MC for $T \geq 100$ K and $B \leq 0.8$ T. The nonzero values of MC are observed even at $T = 300$ K. These are consistent with the results of the main text.

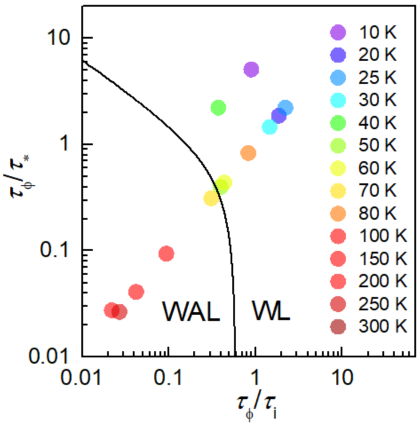

Figure S4. Phase diagram of quantum interference in graphene. The scattering times τ_ϕ, τ_i, τ_*, are estimated by the fitting for the MC at V_g' with Eq. (1) in the main text. The solid line is drawn along Eq. (2) in the main text with $\Delta\sigma_{xx} = 0$.

Figure S4 shows the phase diagram for V_g'. The separation line of WL and WAL regions shows almost the same results as that at the DP. We note that the scattered results for the low-temperature limit can be a signature of electron–hole puddle formation and associated ambiguity in the fitting procedure as noted in the main text.

S3: Temperature dependence of the conductivity and resistivity

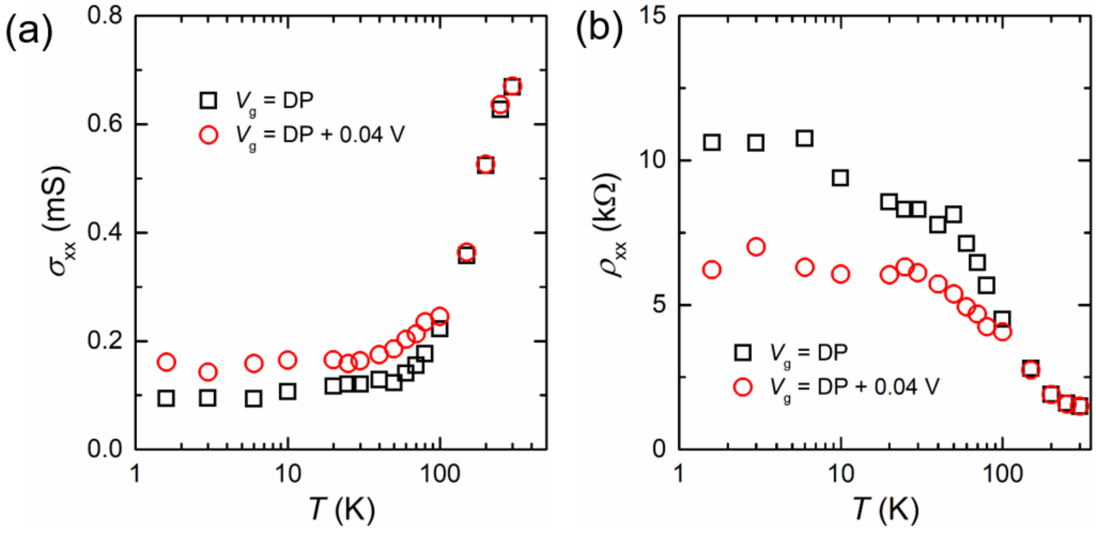

Figure S5. (a) Longitudinal conductivity versus temperature plot of our device at the Dirac point (black) and the electron density of ~7.0 × 10^9 cm^{-2} (red). (b) Resistivity versus temperature plot of the device for the same condition in (a).

Figure S5 shows the longitudinal conductivity σ_{xx} and resistivity ρ_{xx} as a function of T in a logarithmic scale at the DP and n_e ~ 7.0 × 10^9 cm^{-2}. The σ_{xx} and ρ_{xx} at the DP almost saturate for $T \leq 6$ K. For the DP, σ_{xx} and ρ_{xx} are expected to vary with T only for $k_B T > E_{puddle}$ [3], where $E_{puddle} \sim \hbar v_F (\pi \delta n)^{1/2}$ (k_B is the Boltzmann constant $\hbar = h/(2\pi)$, h the Planck constant, v_F ~ 10^6 m/s the Fermi velocity of graphene, δn the carrier density fluctuation). In our device the σ_{xx} saturates ~ 2.5 e^2/h for $T \leq 10$ K, corresponding to the δn ~ 5.5 × 10^7 cm^{-2}. This result confirms the high quality of our device, which is comparable with data in the literature [3].

References in Supplementary Information

1. Morozov, S. V. *et al.*, Strong suppression of weak localization in graphene. *Phys. Rev. Lett.* **97**, 016801 (2006).
2. Beenakker, C. W. J. & van Houten, H. Quantum transport in semiconductor nanostructures. *Solid State Phys.* **1**, 44 (1991).
3. Dean, C. R. *et al.*, Boron nitride substrates for high-quality graphene electronics. *Nat. Nanotechnol.* **5**, 722–726 (2010).
